# Global earth mineral inventory: A data legacy

**DOI:** 10.1002/gdj3.106

**Published:** 2020-11-11

**Authors:** Anirudh Prabhu, Shaunna M. Morrison, Ahmed Eleish, Hao Zhong, Fang Huang, Joshua J. Golden, Samuel N. Perry, Daniel R. Hummer, Jolyon Ralph, Simone E. Runyon, Kathleen Fontaine, Sergey Krivovichev, Robert T. Downs, Robert M. Hazen, Peter Fox

**Affiliations:** 1 Tetherless World Constellation, Rensselaer Polytechnic Institute, Troy, NY, USA; 2 Carnegie Institution for Science, Geophysical Laboratory, Washington, D.C., USA; 3 CSIRO Mineral Resources, Kensington, CBR, Australia; 4 Department of Geosciences, University of Arizona, Tucson, AZ, USA; 5 University of Notre Dame, Notre Dame, IN, USA; 6 Department of Geology, Southern Illinois University, Carbondale, IL, USA; 7 Mindat.org, Mitcham, UK; 8 Department of Geology and Geophysics, University of Wyoming, Laramie, WY, USA; 9 Kola Science Centre of the Russian Academy of Sciences, Apatity, Russia

**Keywords:** data legacy, information model, information system, mineral inventory, minerals, online interface

## Abstract

Minerals contain important clues to understanding the complex geologic history of Earth and other planetary bodies. Therefore, geologists have been collecting mineral samples and compiling data about these samples for centuries. These data have been used to better understand the movement of continental plates, the oxidation of Earth's atmosphere and the water regime of ancient martian landscapes. Datasets found at ‘RRUFF.info/Evolution’ and ‘mindat.org’ have documented a wealth of mineral occurrences around the world. One of the main goals in geoinformatics has been to facilitate discovery by creating and merging datasets from various scientific fields and using statistical methods and visualization tools to inspire and test hypotheses applicable to modelling Earth's past environments. To help achieve this goal, we have compiled physical, chemical and geological properties of minerals and linked them to the above-mentioned mineral occurrence datasets. As a part of the Deep Time Data Infrastructure, funded by the W.M. Keck Foundation, with significant support from the Deep Carbon Observatory (DCO) and the A.P. Sloan Foundation, GEMI (‘Global Earth Mineral Inventory’) was developed from the need of researchers to have all of the required mineral data visible in a single portal, connected by a robust, yet easy to understand schema. Our data legacy integrates these resources into a digestible format for exploration and analysis and has allowed researchers to gain valuable insights from mineralogical data. GEMI can be considered a network, with every node representing some feature of the datasets, for example, a node can represent geological parameters like colour, hardness or lustre. Exploring subnetworks gives the researcher a specific view of the data required for the task at hand. GEMI is accessible through the DCO Data Portal (https://dx.deepcarbon.net/11121/6200-6954-6634-8243-CC). We describe our efforts in compiling GEMI, the Data Policies for usage and sharing, and the evaluation metrics for this data legacy.

## 1 ∣ INTRODUCTION

Due to their chemically and physically robust nature, minerals are the oldest materials on Earth. Consequently, they offer an opportunity to study physical samples that represent Earth and other planetary bodies’ complex geologic past. Minerals form as a result of unique chemical and physical conditions and thereby contain information regarding their formational processes and materials, as well as insights into any subsequent weathering and alteration. Therefore, Earth and planetary science research aims to understand the spatial and temporal diversity and distribution of minerals and their complex relationships with geologic, biological and planetary materials and processes. In order to achieve these goals, scientists study minerals, their geological and biological properties and their occurrence on Earth and planetary surfaces. A mineral occurrence is often referred to as a ‘locality’—a mineral locality is a spatial area in which one or more minerals are found. Mineral localities are user-defined and may correspond to a mineralization event, geologic unit or geographical area. Herein, we treat localities as the spatial area defined in the Mindat database (https://www.mindat.org/). While there are established databases for some of the mineral occurrence information, such as mindat.org, a good portion of the relevant data still reside in scientific publications. In order to create a consolidated data resource, as a part of the Deep Time Data Infrastructure (DTDI) and the Deep Carbon Observatory (DCO), we have developed the Global Earth Mineral Inventory (GEMI), which links the existing databases and adds other relevant data fields from scientific literature. The contributions of this work include (a) providing a single entry point for accessing a set of diverse mineralogical data and databases, (b) an information model that represents the relationships amongst mineralogical data in a schema that is clearly digestible by nondomain scientists, (c) the Mineral Properties Database data collection and development and (d) information extraction performed on the Handbook of Mineralogy collection. Hereafter, Section 1 introduces and details the data resources. Section 2 describes the data collection methods used in the major data resources. Section 3 introduces GEMI’s information model, and Section 4 describes the implementation of this information model.

## 2 ∣ DATA RESOURCES

GEMI was developed by assimilating data from the resources listed in this section and adding data extracted from scientific publications and the handbook of mineralogy. The locality information is compiled from Mindat, and the mineral properties are compiled from associated databases of the RRUFF Project and our parsing of the Handbook of Mineralogy (Anthony et al., 1990-2003; http://www.handbookofmineralogy.org/).

### 2.1 ∣ Mindat.org

Mindat.org is an interactive mineral locality database associated with Hudson Institute of Mineralogy. It currently contains nearly 300,000 mineral localities around the world and on other planetary bodies, including Apollo Lunar samples and meteorites, with over 1 million mineral–locality pairs and nearly 1 million mineral photographs. While the vast majority of the data are retrieved from published literature, mindat.org also provides patrons the opportunity to add localities, mineral–locality pairs, photographs and references. Each of these entries is reviewed by the Mindat team to ensure accuracy and completeness. Mindat treats mineral localities as either a point defined by latitude, longitude and planetary object (if not Earth) or as one or more polygons defining an area on the surface of the Earth or object. In cases where accurate coordinates are not known, they are estimated along with a calculated margin of error. It has become a rich resource for scientific research, and its data have been used in many studies of the diversity and distribution of minerals on Earth's surface (Hazen et al., 2015a,b; Hystad et al., 2015a,b; Hazen et al., 2016; Hazen et al., 2017; Liu et al., 2017a,b; Morrison et al. 2017; Liu et al., 2018; Hystad et al., 2019). These studies include those of carbon mineral ecology (Hazen et al., 2016), which led to the Carbon Mineral Challenge (mineralchallenge.net)—an initiative to engage the scientific and collector community in finding and identifying the 145 ‘missing’ carbon mineral species predicted to currently exist on Earth's surface (Hystad et al., 2019). As of May 2019, the Carbon Mineral Challenge reports more than 30 new mineral species, two of which, parisite-(La) and abellaite, were predicted by Hazen et al. (2016), along with several others of chemical compositions that are very close to predicted minerals (Hummer, 2019). As of the writing of this paper, Mindat data dumps are available by request only. But the Mindat team are working on moving the data to a creative commons licence with an open API for data integration.

### 2.2 ∣ The RRUFF project and associated databases

The RRUFF Project (RRUFF.info) is an ongoing effort to create and maintain a mineral library and databases that provide high-quality chemical, spectral and diffraction data for mineral species (Lafuente et al., 2015), founded and maintained by Dr. Robert T. Downs in the Department of Geosciences at the University of Arizona. The project began out of necessity due to the lack of systematic and freely available Raman spectroscopic data and subsequently grew into one of the most widely used Raman and X-ray diffraction libraries in the world, with more than 780 million downloads from the American Mineralogist Crystal Structure Database (http://rruff.geo.arizona.edu/AMS/amcsd.php; Downs and Halls-Wallace, 2003) alone (as of May 2019). Originally designed to facilitate the crystallographic research of Dr. Downs’ laboratory, the RRUFF Project databases are now used in many facets of Earth, planetary and materials science, including for the NASA Mars Science Laboratory Mission (Bish et al., 2013; Blake et al., 2013; Vaniman et al., 2014; Achilles et al., 2017; Rampe et al., 2017; Yen et al., 2017; Morrison *et al.* 2018a; 2018b; Lewis et al., 2019). The database is continually expanding and currently contains over 10,000 mineral samples and more than 3,500 distinct mineral species. Also associated with the RRUFF Project are additional databases and collections of information, including the Mineral Evolution Database (RRUFF.info/Evolution; Golden, 2019, see below), the interactive list of International Mineralogical Association (IMA)-approved mineral species (RRUFF.info/IMA; see below) and PDFs of the Handbook of Mineralogy, the Canadian Mineralogist, American Mineralogist, Zeitschrift fur Kristallographie and more. All the datasets and publicly accessible journal articles available on the RRUFF Project website are open-access for educational/noncommercial use.^1^ The data resources that are used and linked in GEMI are listed below.

#### 2.2.1 ∣ International Mineralogical Association (IMA) list of approved mineral species

The IMA list of mineral species is found at RRUFF.info/IMA. Users are able to search the list of over 5,000 mineral species (as of January 2019) by name, subset by chemistry, cell parameters and crystallography, or by various tags, including but not limited to structural group, paragenetic mode and availability of crystal structure file. This site also contains useful information about each mineral species, such as chemical composition, oldest known age, structural group name and more—all of which can be downloaded in a number of machine-readable file formats. Additionally, mineral species have associated lists of measured unit cell parameters and compositions, along with links to their information pages in the Handbook of Mineralogy (Anthony et al., 1990-2003; http://www.handbookofmineralogy.org/), measured data in RRUFF Project databases, crystal structure files in the American Mineralogist Crystal Structure Database (AMCSD; Down and Halls-Wallace, 2003), and age and locality data in the Mineral Evolution Database (MED; see below).

#### 2.2.2 ∣ Mineral evolution database

The Mineral Evolution Database (MED;RRUFF.info/Evolution) contains mineral locality and age information extracted from primary literature sources and the mineral–locality database, mindat.org (Golden et al., 2016; Golden, 2019), for the purpose of studying and characterizing mineral diversity and distribution through deep time and its relation to geologic, biological and planetary processes (Hazen et al., 2015a,b; Hystad et al., 2015a,b; Hazen et al., 2016; Hazen et al., 2017; Liu et al., 2017a; Morrison et al. 2017; Liu et al., 2018; Hystad et al., 2019). Currently, the MED hosts 16,553 unique ages for 6,483 directly dated localities, documenting 745,999 mineral–locality pairs and over 210,037 mineral–locality-age triples (as of February 3rd, 2020).^2^ These data have been carefully curated and documented to maximize the accuracy of age associations (between the locality and the minerals), which include data on specific mineral formations, mineralization events, element concentrations and/or deposit formations. The RRUFF website enables many sorting and displaying options, including sorting by maximum or minimum age or alphabetically by locality name and displaying all of the queried minerals at a given locality or displaying a line of data for each mineral–locality pair. Likewise, these data are available for download directly from the website with various user-defined sort, display and file format options.

#### 2.2.3 ∣ Mineral properties database

The Mineral Properties Database (MPD; https://odr.io/MPD) database began in the Deep Time Data Infrastructure (DTDI) project with the goal of better understanding the multidimensional, multivariate trends amongst mineral species and their relationships to geologic materials; geologic, biological and planetary processes; and preservational and sampling biases. As of April 2019, this database contains dozens of parameters associated with copper (Cu) and uranium (U) minerals, including age, colour, redox state, method of discovery, structural complexity and many more, and will be expanded to minerals of each element of the periodic table in the future. These data are the basis for mineral network analysis studies (e.g. Morrison et al. 2017; Perry et al., 2018; Hazen et al., 2019b; Hazen et al., 2019a) and provide a platform for studying the changes in redox conditions through deep time. The Open Data Repository's (ODR; http://www.opendatarepository.org/) interface allows users to tailor their viewing experience as well as subset and sort the data for download (Lafuente et al., 2018). MPD will expand the data parameters available for Cu and U minerals and will scale up to include all known mineral species of every element.

## 3 ∣ DATA COLLECTION METHODS

### 3.1 ∣ Mineral evolution database

The goal of the MED is to associate ages with minerals from around the world and determine the age of each mineral occurrence (Golden, 2019). Mindat.org was used as the source for mineral localities, while the age data have been collected from primary literature as well as pre-existing databases, including many large databases from the United States Geological Survey (USGS), some with thousands of data entries (e.g. Singer et al., 2008, 2009).

The first step in associating ages from the literature with minerals is matching locality names from the source data with Mindat's localities. The primary literature includes journal articles, books, abstracts, dissertations and government open-file reports. In some cases, the locality names in the source data do not match those in Mindat, but instead may refer to geologic features such as intrusions, mountain belts or rock units. In these situations, associating these geologic localities and Mindat's localities is crucial.

Existing databases with ages are matched to mindat localities using both place names and GPS coordinates; however, both are not always available. The North American Volcanic and Intrusive Rock Database (NAVDAT; http://www.navdat.org/NavdatSearch/index.cfm) and the U-Th-Pb and the Rb-Sr sections of the USGS hosted National Geochronology Database (https://mrdata.usgs.gov/geochron/) both contain coordinates but not locality names, so they were matched to the nearest Mindat locality within 1 Km. The USGS also has a variety of commodity databases contained within its Mineral Resource and Online Spatial Databases (MRDATA; https://mrdata.usgs.gov/), which have both locality names and coordinates, so both were considered when matching its data to mindat localities. Note that currently only latitude and longitude are considered, but the managers of MED and Mindat data resources are working to incorporate depth and/or altitude information where possible.

The peer-reviewed geologic literature (e.g. American Mineralogist, Canadian Mineralogist, Journal of Geosciences, Economic Geology) is a robust resource, containing a wide variety of complex, often disparate data. Significant effort is required to collect ages along with relevant geologic context. This context includes the type of material being dated, the geologic event that was dated and whether the age should be associated with the formation of a mineral, an element, a tectonic event, a fluid migration, a cooling, an ore formation or other geological events. Other contextual data collected include any inference or explicit statement by the publishing authors based on their first-hand knowledge of the locality of interest, as well as general information on geologic relationships.

**FIGURE 1:**
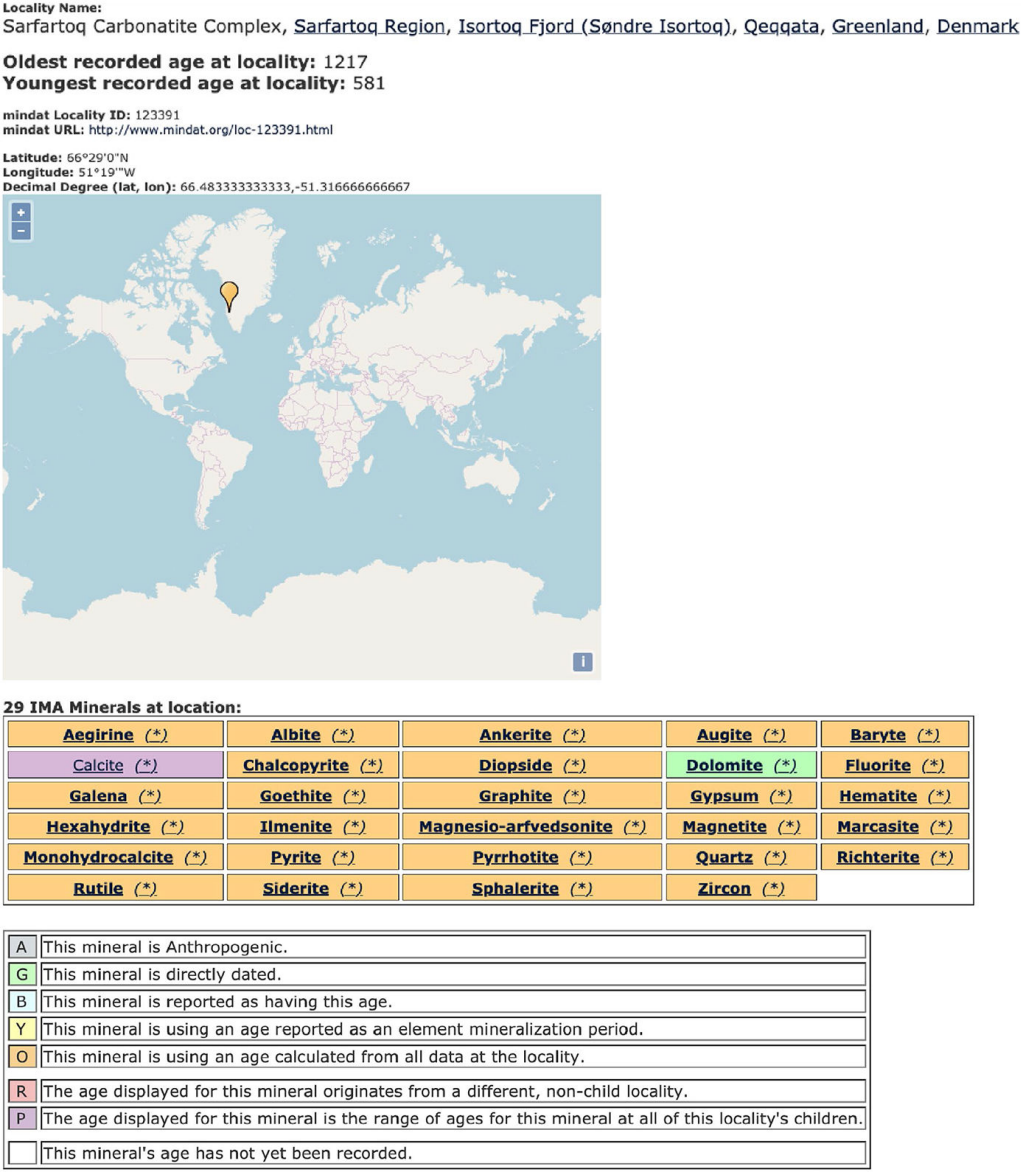

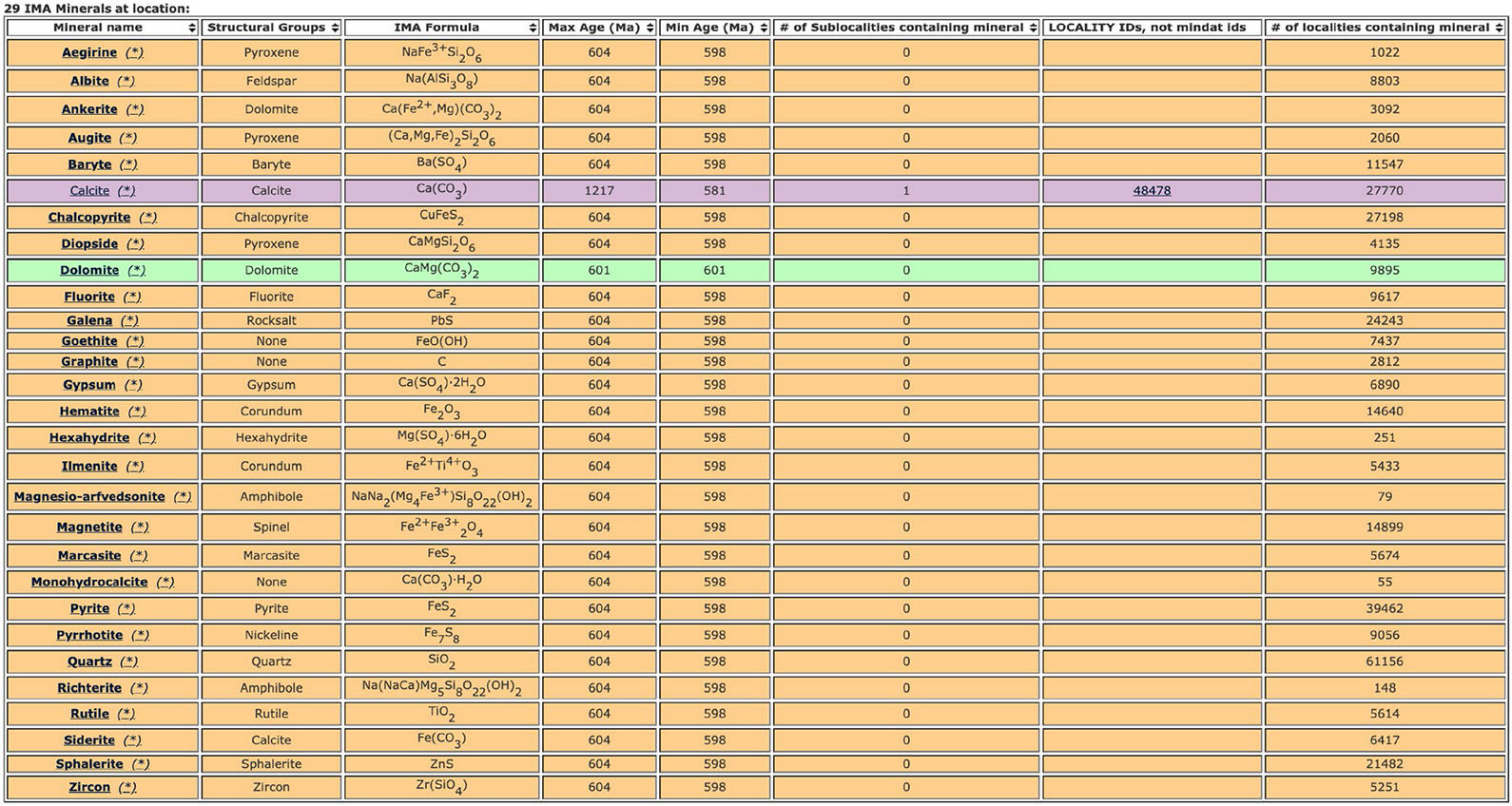
(a) Mineral Evolution Database (MED) locality page and mineral list for Sarfartoq Carbonatite Complex, Sarfartoq Region, Isortoq Fjord (Søndre Isortoq), Qeqqata, Greenland, Denmark (https://rruff.info/mineral_list/locality.php?mindat_id=123391). This webpage contains information on the oldest known and youngest known mineral age at this locality (in millions of years), the locality's Mindat ID and URL, latitude and longitude and the minerals attributed to this locality along with their age classification and associated legend. Dolomite is the only directly dated mineral at this locality and is therefore coloured in green, per the legend scheme. The age of dolomite is assigned to all other minerals at this locality with the exception of calcite. Calcite is assigned the age range of all calcite ages of sublocalities (‘child localities’). (b) Expanded mineral data table of Figure 1a. This expansion gives access to information on ages, structure types, chemical composition and the number of localities each mineral has in MED

Golden (2019) describes the development of an algorithm used to assign an age to each mineral at each locality based on the collected data, as well as inferences and explicit statements by the publishing authors. The data fields relevant to the algorithm include which locality or localities to attribute the age to, which specific mineral was dated and any other minerals or elements that should be assigned the same age. The result is locality–mineral-age triples that are displayed in the MED. Figure 1a shows an example of how an age was assigned to the mineral locality ‘Sarfartoq Carbonatite Complex, Sarfartoq Region, Isortoq Fjord (Søndre Isortoq), Qeqqata, Greenland, Denmark’ (https://rruff.info/mineral_list/locality.php?mindat_id=123391; Lafuente et. al, 2015; Golden et al, 2016; Golden, 2019). Here, dolomite is the only directly dated mineral and, therefore, its age is applied to all of the other minerals at this locality, with the exception of calcite, whose age is classified as the age range of calcite at this locality and all sublocalities (‘child localities’). The user can expand the mineral table to access information on ages, structure types, chemical composition and the number of localities each mineral has in MED.

### 3.2 ∣ Mineral properties database

In the course of the DTDI project, an extensive database of Cu and U mineral properties and characteristics was assembled using the ‘Export Options’ feature of the IMA list of approved mineral species at RRUFF.info/IMA (see above). This feature allowed us to generate a machine-readable, comma-delimited file with the name, chemical composition, list of chemical elements, crystal system, structural group, paragenetic mode, year of first publication and oldest known age from the MED (see above) for each Cu and U mineral species. The ‘Get Locality Counts’ feature was used to extract the number of localities associated with each species from the MED. The structural complexity parameters were calculated using the published crystallographic information files (CIFs) input into the ToposPro software package (Blatov et al., 2000), using methods developed by Dr. Sergey Krivovichev (Krivovichev, 2013, 2016, 2017). Many of the other parameters, such as hardness, colour, lustre, space group and density, were manually extracted from the Handbook of Mineralogy (HoM; Anthony et al, 1990-2003). Associated minerals of each U mineral were compiled from the HoM or the primary literature.

### 3.3 ∣ Parsing the handbook of mineralogy

The Handbook of Mineralogy series (Anthony et al, 1990-2003) is a five volume set authored by John W. Anthony, Richard A. Bideaux, Kenneth W. Bladh and Monte C. Nichols and published by Mineral Data Publishing. Each mineral known at the time of publication occupies one page of the handbook where various pieces of information about the mineral are listed, including its crystal and unit cell data, physical and optical properties, chemistry, occurrence, amongst other attributes. The copyright of the series was given by the authors to the Mineralogical Society of America in 2001 along with PDF files of each page of the handbook. The pdfs were also given to Dr. Downs by Dr. Bideaux, to be freely available to the public through his databases. These files are freely available at the website http://handbookofmineralogy.org/.

**FIGURE 2:**
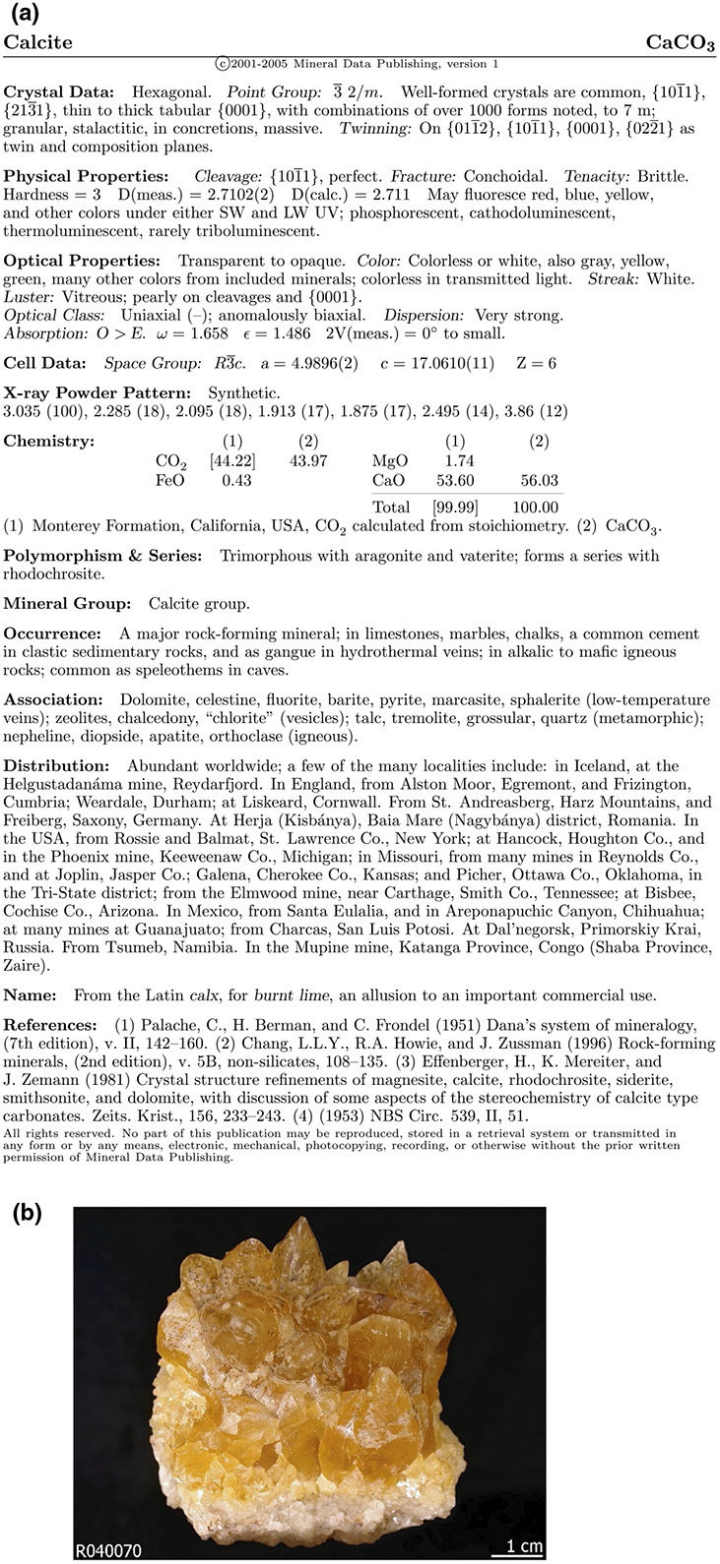
(a) An example of the Handbook of Mineralogy PDFs from which mineralogical information was extracted with automated information extraction (credit: The Handbook of Mineralogy via the RRUFF Project, http://rruff.info/doclib/hom/calcite.pdf). (b), Calcite mineral sample (Credit: The RRUFF Project database, http://rruff.info/calcite/display=default/R040070)

The data contained within the handbook are considered highly useful and relevant to the mineral inventory and as such an effort was undertaken by the DTDI and DCO Data Science teams to programmatically parse and extract a number of attribute values from the PDF files to be incorporated into the inventory. The PDF documents in the Handbook of Mineralogy follow a fixed template to present mineral data features (Figure 2). This makes it fairly straightforward to perform pattern matching on the parsed text to extract data and store into a csv file. For example, we search the parsed text under physical properties for hardness and store calcite's hardness value in the csv file.

These data are then cleaned and uploaded to GEMI by matching the concepts of the information model to the parameters of the data. The code used for information extraction can be found at https://github.com/tetherless-world/GEMI.

## 4 ∣ GEMI INFORMATION MODEL: SPECIFYING THE IMPORTANT ATTRIBUTES FOR EARTH MINERALS AND THEIR RELATIONSHIPS

Information modelling is a technique for specifying the data requirements that are needed with an application domain (Lee, 1999). There are three levels of encoding for information models (ANSI, 1975). Levels of abstraction/encoding represent a different model of the same information and processes, but with increasing amounts of detail.

**FIGURE 3:**
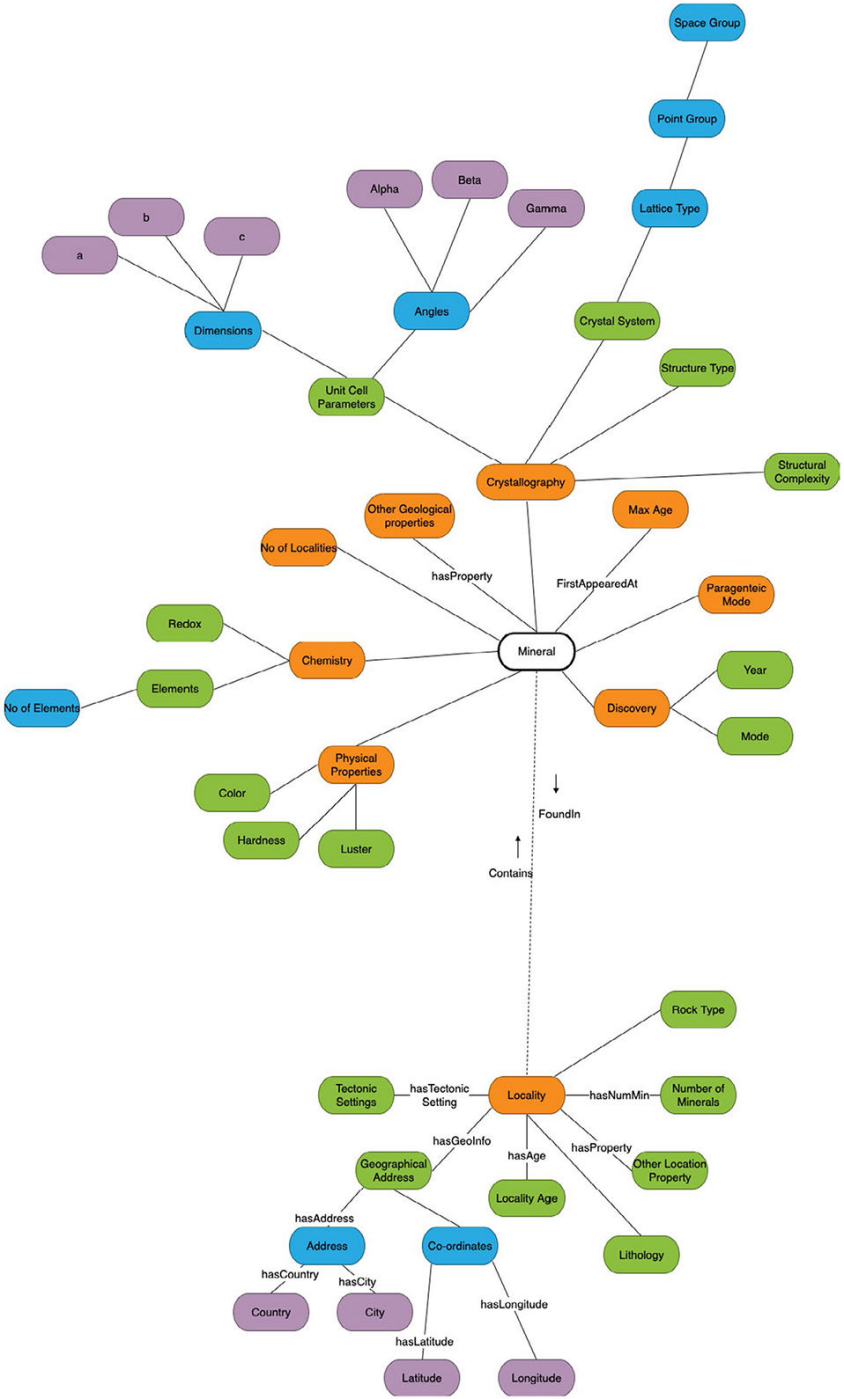
GEMI information model. The information model consists of 2 main parts, the geological properties centred around minerals and the location properties centred around the mineral locality. This is the latest version of the information model as of the publication of this paper

- **Conceptual Models** (or Domain Models) are typically used to explore the concepts and relationships between the concepts in a given domain. The conceptual model is developed to explore high-level structures and concepts. Figure 3 shows the conceptual model for GEMI.
- **Logical Models** typically consist of fully normalized entities (concepts) and meaningful properties (relationships). Ontologies and Entity-Relation Models are examples of logical models. Classes and properties in a logical model must be meaningful and robust enough to meet the requirements of various use cases. For example, the concept Locality Age must accept only numeric data, whereas Country or City may accept any string inputs.
- A **Physical Model** is a single logical model instantiated in a specific information system in a specific installation. Physical Models focus on implementation details and configuration choices for a specific version or product. For example, physical models for GEMI would include index construction, declaring data storage objects, and identifying facets for exploring the data, like filtering results by the presence of a chemical element or by hardness.

The GEMI information model was developed to be a singular schema for all mineral data. It is a robust, yet easy to understand and flexible model. As seen in Figure 3, the GEMI conceptual model is centred around the ‘mineral’. Each mineral has various chemical, physical and geological properties. The properties listed are not meant to be exhaustive, rather they represent the data currently available in the inventory. One of the most important concepts in the model is the ‘locality’. Localities usually have their own set of properties, like geographical address (with the coordinates), tectonic settings, locality age, number of minerals and list of minerals found. The information model presented in this paper provides the concepts from the resources mentioned in Section 2 and relationships between these concepts (properties) and instantiates the model for easy access and retrieval. The information model for GEMI has been designed in a way that allows for partial updates and retrieval. For example, as new data points are added by the original data sources, we add them to GEMI giving the other parameters NA values, until those are populated by the other databases or by a contribution made to GEMI. The GEMI team has developed a set of scheduled maintenance scripts to pipeline the conversion and loading process for the data from these individual data sources. We will also periodically update the GEMI data by processing the data pulled from the original sources.

## 5 ∣ IMPLEMENTATION/METHODS

**FIGURE 4:**
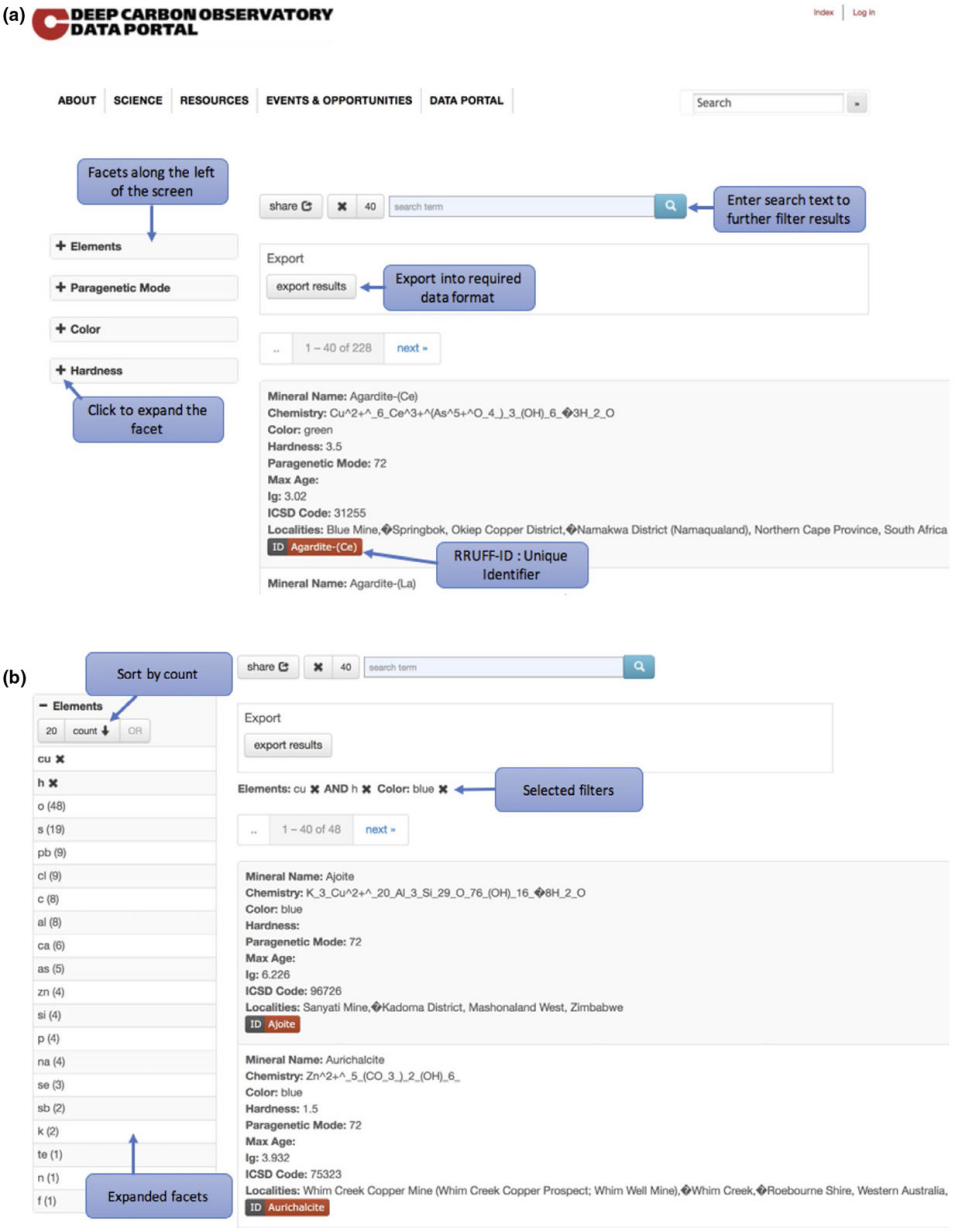
(a) and (b) A screenshot of the user interface is shown in the figure above. A list of results returned on that particular browser appears at the centre and extends down and to the right. On the left-hand side, there is a list of facets, corresponding to properties of the data type to which the results correspond. A user can constrain the criteria that control the query by selecting values within the facets. A feature of the browser is that once a facet value is selected, the result set and all values available in other facets are refreshed after the query is rerun. The user can make selections in several facets at once and can also use the search box above the result set to perform a free-text search. Other actions such as exporting the result set are implemented based on specific requirements for each browser and are highly customizable

As seen in Figure 4, the GEMI interface provides a faceted search interface for the user to query the data and create subsets according to the needs of the use case. Elastic search is the search and analytics engine used to store and query the data, while facetview2 (https://github.com/CottageLabs/facetview2) builds an interface that uses the data in the ElasticSearch Application Programming Interface (API) and allows users to create constraints for a query to be executed.

### 5.1 ∣ Indexed data storage

Elasticsearch is a distributed search and analytics engine that stores data and provides access to the contents through an Application Programming Interface (API). Elasticsearch accepts data in the JavaScript Object Notation (JSON) format, and thus, new data to be ingested into the data store are passed through a processing step to generate a JSON copy. Typically, these data will be in delimited text files where conversion to JSON is done by using a script written in the Python programming language. In addition to the conversion operation, the script places the output in the structure required by the Elasticsearch Bulk API which is responsible for importing data in bulk into the data store. Given the diverse nature of sources of incoming data into GEMI and their multiple formats, a detailed discussion of the processing required for conversion is beyond the scope of this paper. Additional information relevant to the processing of data from the major resources can be found on the GEMI github repository (https://github.com/tetherless-world/GEMI).

### 5.2 ∣ Semantically enriched faceted browser

We also developed a faceted browser interface to facilitate access to the data residing in Elasticsearch. A faceted search allows the user to narrow down search results (in this case mineral samples and localities) by applying multiple filters based on the features seen in the data (Tunkelang, 2009). This web-based interface was built using Hyper Text Markup Language (HTML) and JavaScript and can be accessed through a web browser. The interface makes use of the JavaScript library FacetView2 (https://github.com/CottageLabs/facetview2), which acts as a bridge to the Elasticsearch API handling the querying and filtering of data. Additionally, the JavaScript libraries JQuery (https://jquery.com/) and handlebars (http://handlebarsjs.com/) are employed to facilitate the coding process by providing templating and dynamic generation of user interface elements as well as extensibility of the code.

GEMI is housed at the DCO Data Portal, which was also built using components similar to those in the DCO Data portal (Ma et al, 2017). The data portal is a collection of data files, and it focuses on presenting the metadata related to dataset and allows the user to download entire datasets for analysis. GEMI on the other hand focuses on enabling the user to browse and filter data with finer granularity. As the name suggests, the ‘Global Earth Mineral Inventory’ information system revolves around ‘Earth Minerals’ but another way to access the data is through the various localities where minerals can be found. The GEMI interface is comprised of 2 data browsers:

- Mineral Browser: The mineral browser is the main entry point to the inventory. This browser presents a faceted view of the mineral data. The data represented in the mineral browser can be accessed and retrieved using a faceted browser. The user can choose a value or a set/range of multiple values to retrieve a subset of minerals in the inventory. The result will be a dataset of the minerals and geological properties required for the current scientific exploration. This dataset can be exported and downloaded as a json or a CSV (comma-separated value) file.
- Mineral Occurrence Browser/Locality Browser: The mineral occurrence/locality browser contains data pertaining to mineral localities. This browser lets the user query mineral localities based on their geological, temporal or spatial properties. The result of the query will be a dataset of localities, minerals found in those localities and any other parameter chosen by the user. This dataset can be exported and downloaded as a json or a CSV file.

The ‘mineral name’ is considered the unique ID to connect the 2 data browsers. Since GEMI provides user with data from multiple sources, using the export functionality on the GEMI browser outputs data files that can be directly used for analysis and to produce visualizations. The information model (described in Section 3) can be extended when additional geological properties or concepts are added.

## 6 ∣ DATA SHARING, USAGE AND PUBLISHING

GEMI presents scientists with continually updated mineral data resources. The users can access and retrieve a subset or all of the data from the data service hosted on the DCO Data Portal. The data in the inventory have been collected, processed and maintained by authors of this paper and their respective organizations. As a DCO data legacy, GEMI follows all the DCO data and information policies (https://deepcarbon.net/page/dco-open-access-and-data-policies). The data contributed to the inventory is in the public domain and thus can be shared and used by any person or organization. The data retrieved from the resources like RRUFF and mindat. org follow the usage policies/licences listed on their respective websites.

Usage of data retrieved from GEMI should be accompanied by formal citation of the dataset and acknowledgement of DCO as a digital community resource. The GEMI data users are also urged to make reasonable and timely efforts to notify the authors of this paper and/or data managers of GEMI of any errors, limitations or problems encountered while using the service or while analysing the data.

### 6.1 ∣ Citation policy

Formal data citation emerged in the 1990s as a method to reward data publishing and sharing by giving credit to contributors, but this has not provided a strong incentive to share data and as a result much of the data lies hidden in the unindexed deep web (Parsons and Fox, 2014). Data citation is an evolving but increasingly important scientific practice to help ensure reproducibility and fair credit for scientific effort. The premise is that a dataset be cited as a first-class scientific object much like an article or book. Citing data is now recognized as one of the key practices leading to recognition of data as a primary research output (Australian National Data Service, n.d.). Martone (2014) lists the data citation principles described by FORCE11. These principles cover the purpose, function and attributes of citation by recognizing the dual necessity of making citation practices that are human understandable and machine actionable (Martone, 2014). Citing a dataset involves many of the components that can be seen in citation literature, for example Authors (or contributors), Title, year of publication, archive location or publisher, version and persistent identifier.

The DCO Data Portal uses the global handle system (https://www.handle.net/) to assign a unique identifier called DCO-ID to the records in the DCO Knowledge Network (Ma et. al, 2017). As a DCO dataset, GEMI is assigned a DCO-ID to be cited when researchers use the GEMI portal to access datasets. GEMI has been created by linking data from various sources; therefore, each original data source has also been assigned a DCO-ID for citation purposes. A record of the DCO-IDs can be found on the ‘Citations’ page of the GEMI browser. GEMI users are required to cite the dataset as follows^3^:

Prabhu A, Morrison SM, Eleish A, Zhong H, Huang F, Golden JJ, Perry SN, Hummer DR, Ralph J, Runyon SE, Fontaine K, Krivovichev S, Downs RT, Hazen RM, Fox P (2019) Global Earth Mineral Inventory: A Deep Carbon Observatory Data Legacy. Version 1.0. DCO. (https://dx.deepcarbon.net/11121/6200-6954-6634-8243-CC).

Usage involving other concepts mentioned in this paper, such as the information model, evaluation metrics or implementation methods, should cite this publication.

### 6.2 ∣ Contributing to GEMI

The data available in GEMI at the time of this publication have been collected and compiled by the authors. While we intend to improve both the completeness and quality of the data and information model, contributions to the inventory are welcome. Contributors must acknowledge that the data they contribute will be openly accessible through GEMI. Contributors will be acknowledged for their work on the DCO Data Portal and GEMI information page. Data contributors are also urged to provide information about their data collection methods and any relevant metadata about the contribution. Data curators from the GEMI Team will then assess whether the contribution can be assimilated into the GEMI information model and contact the contributors with suggestions. Every contribution made to GEMI will be assigned a DCO-ID so that its usage and potential impact can be tracked, while also giving credit to the contributors of the data.

## 7 ∣ EXAMPLES OF ACCESS AND USE

### 7.1 ∣ Copper

Copper was one of the first metals to be mined and used by ancient peoples, effectively bringing human civilization out of the Stone Age and into the Bronze Age. Today, native Cu and its alloys, like bronze (a mixture of Cu and Sn, often with other metals), are commonly used in many industrial applications, including electrical wiring, plumbing, transportation and door knobs and hand-railings, due to its unique traits of malleability, electrical conductivity, corrosion resistance and antimicrobial properties. Cu atoms are also utilized by life—oxidoreductase proteins bind Cu into their active sites to support their functions, such as haemocyanin, which uses Cu to oxidize the blood of certain invertebrates with the same Fe-based mechanism that haemoglobin employs to oxidize the blood of humans and most vertebrates (Coates and Nairn, 2014). With such a wide range of uses, both industrial, biological and in between, Cu is of interest to a broad swath of scientific researchers. Our particular scientific interests are in better understanding Cu mineral occurrence and the geologic and geochemical processes associated with deposit formation, characterizing the relationship between Cu minerals and all other mineral species in order to develop predictive algorithms for locating previously unknown mineral locations and in constraining the bioavailability of Cu^2+^ through deep time in relation to the emergence and evolution of Cuutilizing proteins. With these objectives in mind, GEMI has amassed a wealth of Cu mineral data and made them available to the scientific community. These data include mineral deposit location, age and mineral assemblages, as well as the physical and chemical properties of Cu-containing mineral species. There are currently 742 (http://rruff.info/ima/; 10 May 2020) mineral species containing Cu in their IMA-approved chemical formulas, with the minerals chalcopyrite (CuFeS_2_) and malachite [Cu_2_(CO_3_)(OH)_2_] having the greatest number of copper-containing localities known on Earth's surface. Early results with these data show that there are strong correlations between Cu redox state and colour; likewise, there are also significant correlations between colour and grain size in relation to the year and method of discovery—hinting at the human sampling bias of mineralogical data (Hazen et al., 2015b; Morrison et al. 2017). Additionally, the network topology of Cu mineral occurrence is heavily segregated by composition, the presence or absence of sulphur and oxygen, specifically (Morrison et al. 2017). Furthermore, preliminary examination of the Cu co-occurrence network reveals an embedded trendline of structural complexity. Future work will predict the location of previously unknown Cu-mineral deposits, constrain the tectonic parameters associated with Cu deposit formation and relate the timing of bioavailability of Cu^2+^ to Earth's coevolution with life.

### 7.2 ∣ Uranium

Uranium is a heavy, radioactive element critically important in energy production and to national security. The most common form of U in the crust is the mineral uraninite, UO_2+x_. When exposed to ambient surface conditions, typically through mining activities, U(IV) readily oxidizes to U(VI) and forms the dioxo uranyl ion, UO_2_^2+^ (Finch and Murakami, 1999). More than 290 minerals contain essential U in their crystal structures, with most containing U(VI) due to the relatively high solubility of U(VI) phases and the uranyl ion readily forming aqueous complexes with oxyanions (Finch and Murakami, 1999). U(VI) minerals are chemically and structurally diverse due to the electronic properties of the uranyl ion (Plášil, 2014). New U minerals are described annually with most resulting from ongoing oxidation–hydration dissolution of uraninite in defunct mines. Due to their importance in controlling U transport, understanding the formation mechanisms, determining the thermodynamic properties and predicting the occurrences of uranium minerals is of scientific interest. To that end, a database of all currently described U minerals has been developed and added as part of GEMI. Properties recorded include crystallographic parameters, thermodynamic data, crystal complexity and associated minerals for network analysis. Early results with force-directed bipartite diagrams of U minerals with their composition appears to show a segregation of mineral groups that form under similar geochemical conditions, such as uranyl phosphates and arsenates, and mineral groups with similar solubilities, such as the uranyl carbonates and sulphates (Finch and Murakami, 1999; Plaášil, 2014; Perry et al., 2018). Cluster analysis between uranium mineral crystal complexity, calculated from crystallographic information files (CIFs) with no H atoms present, suggests possible correlations between minerals of similar structural unit topologies and structural types. Future work will attempt to derive predictive models for uranium mineral occurrence based on their associated minerals.

### 7.3 ∣ Manganese

Manganese (Mn) is the tenth most abundant element in Earth's crust, an essential component of over 600 mineral species, with three different naturally occurring oxidation states (+2, +3, +4), making it an ideal proxy for the redox state of Earth's crust. An analysis of the frequency of co-occurrence of Mn minerals enables the creation of a network diagram showing clusters of Mn minerals that tend to form together. This analysis yields three distinct clusters: (a) a central cluster composed of primary Mn^2+^ silicates, oxides and carbonates formed in igneous intrusions, hydrothermal ores and skarns, together with secondary Mn^3+^ and Mn^4+^ oxidative weathering products, (b) a side cluster of predominantly Mn^2+^ phosphates and oxides arising in granitic pegmatites and (c) a side cluster of predominantly Mn^2+^ silicates arising in highly alkaline, agpaitic intrusive rocks (Hummer et. al, 2018). Clusters 2 and 3 display some co-occurrence with minerals in cluster 1, but not with each other (Hummer et al., 2018).

Ages of first appearance of the minerals in these clusters reveal that common, primary igneous and metamorphic minerals of clusters 1 and 2 appear early in the geologic record, at or before 2.0 Ga. Oxidative weathering products in cluster 1 predominantly appear after photosystem-II initiated the Great Oxidation Event at ~2.3 Ga. Minerals of cluster 3 are geologically recent, appearing in the last 1.2 Ga as a result of further reworking and differentiation of the crust. Knowing the oxidation state of Mn in every individual occurrence also enables the calculation of the average Mn oxidation state across geologic time. Although older data do not reveal clear trends that overcome statistical errors, data from the last 600 Ma reveal a steadily increasing proportion of Mn^4+^ minerals and an increasing average oxidation state. Strikingly, the trend in average Mn oxidation state exactly mirrors reconstructions of atmospheric oxygen (Berner and Canfield, 1989; Bergman et al., 2004; Holland, 2006), but with a time lag of 66 Ma. This correlation indicates that oxygenation of the atmosphere during the Phanerozoic drove the oxidation of Mn in Earth's crust but required on average 66 Ma for various geologic processes to rework and expose crustal material to the increasingly oxygenated atmosphere. These results also suggest strategies for using other mineral proxies to place constraints on the redox state of other Earth reservoirs across geologic time.

### 7.4 ∣ Other applications

Other applications that used data from GEMI include Moore et al. (2018), Liu et al. (2017a; 2019), Grew et al. (2019) and Ostroverkhova et al. (2017, 2019). Moore et al. (2018) used cobalt minerals to create an evolving network highlight how the cobalt mineral environments evolved over time. Co is a minor component of basalt and the weathering of basalt was a consistent trace source of Co to the ocean. Because Co does not form many minerals, especially in the Archean Eon (before 2,500 Ma) Co could be weathered from basalt. After 2,500 Ma, more oxygen containing Co minerals form showing the influence of global oxygenation on mineralogy. Liu et al. (2017a) investigated the trends in mineral occurrence by element through deep time and in doing so recognized that mineral occurrences associated with the Rodinian supercontinent assembly show a markedly different signature than other supercontinent assemblies. Specifically, most minerals have relatively low abundances with the exception of those associated with Nb, Y and Zr, which are enriched and more abundant than originally expected. This trend in mineral occurrence led the authors to explore the geochemical data available at EarthChem.org in order to fully characterize the mineralogical and geochemical profile of Rodinia and its relationship to other supercontinents. This study has spurred ongoing investigations into the underlying causes for the unexpectedly different mineralogy and geochemistry in the Rodinian supercontinent (Liu et al., 2019). Ostroverkhova et al. (2017, 2019) explored the multidimensional co-occurrence relationships between Li-bearing minerals. These studies found distinctly different clusters of Li minerals which correspond to the composition of the source magma and allow distinction between parental source material based on Li mineral assemblage.

## 8 ∣ CONCLUSION AND FUTURE WORK

The Deep Carbon Observatory (DCO) provides a collective resource to facilitate discovery, understanding and collaboration across disciplines and generations (https://deepcarbon.net/page/dco-open-access-and-data-policies). The Global Earth Mineral Inventory can be accessed as a part of the DCO Data Portal. Long-term goals of GEMI include not only continued storage of the data, but also easy access and use of these data. The GEMI information system is flexible and can be extended to accommodate contributions from the geoscience community. Future improvements for GEMI include adding network and map visualization features linked to the data and conducting usability testing with scientists using the evaluation method developed by the authors of this paper.

We have developed a method to evaluate a data legacy based on qualitative and quantitative metrics that measures the value and impact of a data resource (Thomson and Fearn, 1996; Pipino et. al, 2002; Charter, 2020). We have also introduced a set of metrics based on the concept of software usability testing (Nielsen, 1994) and have adapted them to apply to data resources such as GEMI. The idea behind usability testing involves users of the data legacy testing the service and using the data retrieved for analysis. The GEMI team plan to implement selected metrics into the GEMI browser. Users will then be able to view the ‘quality’ of various contributions made to GEMI. The method and metrics for evaluating a data legacy are stated below. This evaluation method, including the usability tests, has not been implemented at this time. The results of our evaluation will be published on the GEMI website after usability tests have been conducted.

### 8.1 ∣ Evaluation metrics

It is hard to evaluate how valuable a data legacy is, since there is not a single standard approach on evaluating a data legacy. Datasets are created to be used by anyone from a single researcher to large communities and the way they use the dataset and what they gain from this is subjective. This makes evaluating them an extremely complex endeavour. In this section, we evaluate DCO data legacies based on a combination of qualitative and quantitative metrics based on (Thomson and Fearn, 1996; Pipino et. al, 2002; RDA Publishing Data Interest Group).

#### 1. Completeness

While quantitative metrics help understand and evaluate a data legacy, it is important to acknowledge that a data legacy is never complete. We iteratively build on existing data resources and add new observations and features to improve existing records. As a result, building a data legacy is an exercise in pursuing the seemingly impossible ‘perfect’ or ‘complete’ dataset. But pursuing a complete data resource leads to further discoveries of important observations and important concepts that are required in a continuously evolving field.

‘Completeness’ for data legacies in the DCO Data Portal is a measure of the average number of observations available in the data across all features. This metric is meant to give the user an estimate of how many observations have been recorded in the data legacy. When accessing a subset of GEMI, the user will be presented with the completeness for that subset of data. As a new attribute/geological feature and its respective observations are added to the data legacy, the completeness metrics may undergo drastic change. This metric helps understand how much missing data a legacy contains, at any given level of granularity. For example, the current version of GEMI records retrieved from MPD is 80.08% complete, while the completeness for Handbook of mineralogy is 77.31% and MED is 29.68%.

#### 2. Fitness for purpose

Fitness for purpose is a ‘property of the data produced by a measurement process that enables the user of the data to make technically correct decisions for a stated purpose’ (Thompson and Fern, 1996). Fitness of purpose metrics tends to focus on the ability of the user to ‘use’ the data service for its originally intended purpose. But for data legacies, like those present in the DCO Data Portal, the focus is to use datasets from various sources and apply truly multidisciplinary methods to achieve one's research goals. This means that the GEMI data legacy (amongst others) can be utilized in a completely different way than the data producers or data stewards intended. For this reason, the authors rely more on ‘Fitness for Use’ as a metric to evaluate the data legacy.

#### 3. Fitness for Use

Fitness for Use focuses on the usability of a product, not only for its originally intended purpose but also for any new use cases or needs that may arise in the future. In the case of a data legacy, usability/fitness for use focuses on how easily the data retrieved from GEMI can be used for analysis. Factors like the machine readability of the data, the file type of the exported data and the data structure of the inventory play an important role in determining the fitness for use.

#### 4. Usability Testing

The concept of usability has been commonly applied to user interfaces and software products. The usability of a data legacy may be similar to the usability of a software or a web service in some ways, but there are some additional considerations to be made while testing the usability of a data legacy. Nielsen (1994) describes usability as a multidimensional property, traditionally associated with 5 attributes. We adapt the definitions of these attributes to apply to data legacies and add additional attributes (highlighted with a ‘*’) that are essential specifically for usability testing of data legacies based on what we find are basic requirements for consumer of any dataset.

- Learnability: Using the data service should be intuitive and easy to learn. Users should not feel burdened by the interface and should be able to retrieve data with ease.
- Efficiency: Data legacy users should not need to spend a lot of their time trying to procure relevant data, and they should instead focus their efforts on using the data to make scientific discoveries. Data legacies should enable such efficient retrieval and usage of data.
- Errors: Data legacies should minimize errors, thereby preventing inaccurate analysis and wrongly drawn conclusions.
- Satisfaction: The user should be satisfied with the data in the inventories, the user interface for the data service and the metadata.
- Readability*: Machine readability is one of the most important characteristics for a data legacy. However, datasets that try to push too hard for machine readability tend to lose human readability. Ideally, a data legacy should try to achieve a balance between the two.
- Analysability*: Machine readability is just the first step in order to use data effectively. Another important attribute is the ‘Analysability’ of the data. Analysability focuses on the ability of the data to be used for various kinds of analysis. Consider the example of datasets that are highly machine-readable, well maintained and documented, but in which the data for some features are very sparse. Sparse datasets, while clean and machine-readable, may prove ineffective for analysis. This can be avoided by iteratively improving the observations in the data, by imputing or deriving certain observations using a statistical or analytical method.
- Entropy*: ‘In information theory, entropy quantifies the amount of uncertainty involved in the value of a random variable or the outcome of a random process’ (Ghahramani, 2006). Data in an inventory should contain metadata for all the attributes. Any other information relevant to the dataset should also be presented to the user, so as to reduce uncertainty.

The evaluation metrics presented in this paper can be applied to data legacies and inventories. Depending on the type of inventory/legacy some combination or all of the metrics can be used to evaluate it.

### 8.2 ∣ Impact

The impact of developing and maintaining a data legacy like GEMI is intended to greatly reduce the data collection and processing efforts for scientists, thereby enabling them to focus on analysis and discoveries. In order to continue the process of developing and maintaining data legacies in general, there must be incentives and due credit given to the individuals and organizations that both develop and contribute to data legacies, and these accomplishments must be viewed as vital improvements to the scientific field, similar to scientific publications.

## Footnotes

1 Terms and conditions found at https://rruff.info/

2 https://rruff.info/mineral_list/MED_summary.php

3 The bib file found on the GEMI GitHub page (https://github.com/tetherless-world/GEMI) can be used to cite the data in other citation styles.
